# Poor adherence to the malaria management protocol among health workers attending under-five year old febrile children at Omdurman Hospital, Sudan

**DOI:** 10.1186/s12936-015-0575-9

**Published:** 2015-01-28

**Authors:** Jalal A Bilal, Gasim I Gasim, Mohamed T Abdien, Khalid A Elmardi, Elfatih M Malik, Ishag Adam

**Affiliations:** College of Medicine, Qassim University, Qassim, Saudi Arabia; Faculty of Medicine, University of Khartoum, PO Box 102, Khartoum, Sudan; Federal Ministry of Health, Khartoum, Sudan; Ministry of Health, Gezira State PO Box 492, Medani, Sudan

**Keywords:** Malaria, Treatment, Diagnosis, Children, Sudan

## Abstract

**Background:**

In spite of the World Health Organization recommendations for the treatment of malaria, febrile patients are still infrequently tested and erroneously treated for malaria. This study aimed to investigate the adherence to malaria national protocol for the management of malaria among under five years old children.

**Methods:**

A cross sectional hospital-based study was conducted during the period from September through December 2013 among febrile children below the age of five years attending the outpatient department of Omdurman Children Hospital, Sudan. Demographic, clinical and laboratory data [blood film, rapid diagnostic test (RDTs), haemoglobin, WBCs and chest X ray] and anti-malarials and/or antibiotics prescription were recorded.

**Results:**

A total of 749 febrile children were enrolled. The mean (SD) age was 37.51 (41.6) months. Less than a half, (327, 43.7%) of children were investigated for malaria using microscopy (271, 82.9%), RDT (4, 1.2%) or both (52, 15.9%). Malaria was not investigated for more than a half, (422, 56.3%) however investigations targeting other causes of fever were requested for them. Malaria was positive in 72 (22%) of the 327 investigated children. Five (1.6%) out of 255 with negative malaria tests were treated by an anti-malarials. Quinine was the most frequently prescribed anti-malarials (65, 72.2%) then artemisinin-based combination therapy (ACT) (2, 27.8%). The majority of the 749 children (655, 87.4%) were prescribed an antibiotic.

**Conclusion:**

There is a poor adherence to malaria management protocol in Sudan among physicians treating children below five years of age. There was a high rate of antibiotic prescription needs.

## Background

In spite of many measurements, malaria remains a big public health problem where it is estimated that about a million deaths and over 400 million malaria cases occur worldwide each year, with 90% of these deaths occurring in sub-Saharan Africa [1]. Owing to the spread of *Plasmodium falciparum* resistant strains in the majority of malaria endemic countries, the World Health Organization (WHO) recommended artemisinin-based combination therapy (ACT) for the treatment of uncomplicated *P. falciparum* malaria, which is adopted in most of the African countries including Sudan [2]. In 2010, the WHO expanded its recommendations to include laboratory confirmation with microscopy or a rapid diagnostic test (RDT) before initiating anti-malarial therapy [3]. Hence, the management of fever in African countries underwent a radical change in the recent years from the presumptive diagnosis and treatment of fever as malaria to formal guidelines [3-5]. Due to the spread of multidrug resistant *P. falciparum* strains in Sudan, the drug treatment policy for uncomplicated *P. falciparum* malaria was changed in the year 2004 from chloroquine to ACT, where artesunate plus sulphadoxine/pyrimethamine (AS + SP) and artemether plus lumefantrine (AL) is the first- and second-line treatment for uncomplicated *P. falciparum* malaria, respectively [6,7]. Although diagnostic tests are available, febrile patients are still infrequently tested for malaria [8,9]. A considerable number of patients are treated for malaria despite negative test results and high a frequency antibiotic prescription for those patients was reported as well [10-12]. In this context, it is vital to investigate the prescription practices of health care providers in Sudan for reasonably accurate information about the causes of childhood death is part of the Target of Millennium Development Goal 4 [13]. Furthermore, information on success/failure of the national malaria programmes in endemic countries will help augment the new plan of the WHO towards malaria elimination since one of operational requirements for elimination is “all malaria cases are microscopically confirmed and treated according to national policy” [14]. The aim of this study was to investigate the adherence to malaria national protocol for the management of malaria among under five years old children.

## Methods

A hospital-based cross sectional study was conducted in Omdurman Children Hospital, during the period from September through December 2013. Omdurman is one of the three cities of the tripartite metropolitan capital, Khartoum. Malaria transmission in Khartoum is recognized as low in intensity [15]. Medical doctors (graduate of medical colleges) are managing children at OCEH are residents who are enrolled in postgraduate training supervising general practitioners who completed more than one year after internship and house physicians or interns who are doing their assessment for permanent registration as general practitioners. The hospital is equipped with a well-prepared laboratory with malaria diagnosis facilities; thin and thick blood films and RDT performed by well-trained technicians.

Population is children under the age of five years who presented with fever to the outpatient/emergency clinics at OCEH. It was assumed that the proportion of febrile under-five year old children who had to receive prescriptions in accordance with the guidelines of treatment of malaria was not known before the study. However, a random sample of 740 children was selected to calculate the proportion of febrile children within 3 percentage points of the true proportion, assuming the true proportion was 70% and that 10% of children would not be respond or have complete data.

During the study period all febrile children ≤5 years of age were included. Using a structured questionnaire, information on age, gender, whether the child complained of cough, runny nose, difficulty of breathing, vomiting, diarrhea, convulsion and inactivity were prospectively recorded at recruitment. A general practitioner or a house physician initially assessed children. Axillary temperature was recorded. Whether, microscopy for malaria, RTD white blood cell count and the chest X-ray were requested and the results, if available, were recorded. Finally, the treating physicians’ prescriptions of anti-malarials and/or antibiotics were recorded.

### Ethics

Children’s guardians were informed about the purposes of the study and they signed a written informed consent prior to enrolment. The study received ethical approval from the ethical committee of the hospital.

### Statistics

Data were entered into computer using SPSS for Window, version 20 (SPSS Inc., Chicago, Illinois). The mean and standard deviation were calculated for numerical data and frequency distribution was calculated for nominal or ordinal variables. Univariate and multivariate logistic regressions were used where anti-malarials and combined anti-malarials/antibiotics prescriptions were dependent variables and demographic variables age and gender, clinical variables and laboratory values were the independent variables. The results were expressed as odds ratios (ORs) and 95% confidence intervals (95% CIs). A p value < 0.05 was considered significant.

## Results

A total of 749 febrile children at the mean (SD) age of 37.51 (41.68) months were enrolled to the study. Males/female ratio was 1.35 [430, (57.4%) vs. 319 (42.6%)]. Table 1 shows the most frequently encountered symptoms beside fever and the laboratory characteristics requested by the treating clinicians.

**Table 1:** **Frequency (%) of symptoms encountered and requested laboratory characteristics in febrile children below 5 years age at Omdurman Hospital, Sudan (N = 749)**

**Variable**	**Frequency (%)**
**Symptoms**	
Cough	398 (53.4)
Shortness of breathing	211 (28.2)
Vomiting	151 (20.2)
Convulsion	103 (13.8)
Diarrhoea	97 (13.0)
Inactivity	30 (4.0)
Runny nose	28 (3.7)
**Laboratory characteristics**	
Blood film for malaria	322 (42.9)
Leucocytes	415 (55.3)
Haemoglobin	313 (41.7)
Chest X-Ray	133 (17.8)
Rapid diagnostic test	66 (8.8)

Around two fifth (327, 43.7%) of these children were investigated for malaria using microscopy (271, 82.9%), RDT (4, 1.2%) or both (52, 15.9%). Other investigations were requested beside malaria for 96 (12.8%) children, these were; white blood cell count (53), haemoglobin (41) and chest X-ray (2). Malaria was not investigated for over half 422 (56.3%) of the children however, haemoglobin concentration, white blood cell count and chest X-ray were requested for (313, 74.2%), 415/422 (415, 98.3%) and (44, 10.4%) of these 422 children, respectively. Malaria was positive in 72 (22%) of the 327 investigated children, 63 of them were positive by microscopy, four by RDT and five were by both.

**Figure 1:**
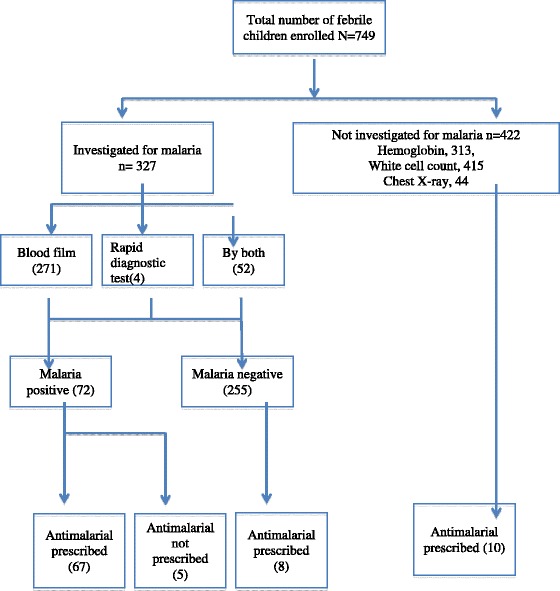
**Febrile children management flowchart at Omdurman Hospital, Sudan.**

The majority of (67, 93.1%) of malaria positive 72 children received anti-malarials whereas, five (6.9%), were not. Malaria was negative in 255 (78%) of the 327 tested children however, 5 (2%) of them received anti-malarials. Among children who were not tested for malaria (n = 422) but tested for other causes of fever, 10 (2.4%) were presumptively treated as malaria (Figure 1).

Quinine was the most frequently prescribed anti-malarial (as intravenous infusion) for 65 (72.2%) cases, followed by AS+ SP and AL for 25 (27.8%) cases. Forty-two (64.6%) out of 65 patients who were treated by quinine, had no any sign of severe malaria.

In a logistic regression, while none of the demographic and clinical characters associated with physicians’ anti-malarials prescription, investigating malaria (via blood film/RDT) was the only variable associated with physicians’ anti-malarials prescription (Table 2). The variables age, cough, breathlessness, vomiting, seizure, the white cell count and microscopy requests were associated with combined anti-malarial/antibiotic prescription however when adjusting for all variables in a multivariate model, only age, vomiting, seizure, white cell count and haemoglobin level were associated the combined anti-malarials/antibiotics prescription (Table 3).

**Table 2:** **Demographic and laboratory characters associated with anti-malarial prescription for febrile less than 5-year-old children at Omdurman Hospital, Sudan using univariate and multivariate analyses**

**Variables**	**Univariate**			**Multivariate**		
	**OR**	**95% CI**	**P value**	**OR**	**95% CI**	**P value**
Age	1.02	1.01-1.02	<0.001	0.98	0.95- 1.01	0.159
Gender	1.34	0.86-2.08	0.199	0.32	0.07- 1.48	0.144
Cough	0.26	0.16-0.43	<0.001	0.59	0.11- 3.10	0.538
Runny nose	1.23	0.42-6.63	0.707	5.76	0.03- 1016	0.506
Breathlessness	0.22	0.10-0.46	<0.001	0.96	0.09- 10.21	0.973
Vomiting	2.1	1.29-3.39	0.003	1.09	0.25- 4.82	0.905
Diarrhoea	0.81	0.41-1.64	0.573	1.43	0.19- 10.64	0.725
Seizure	1.98	1.15-3.42	0.014	5.53	0.89- 34.2	0.066
Lethargy	1.46	0.90-2.35	0.122	0.66	0.03- 16.99	0.801
Temperature	1.00	0.88-1.13	0.998	1.14	0.61- 2.11	0.691
Leucocytes	1.00	1.01-1.05	0.036	1.00	0.99- 1.00	0.991
Chest X-ray	0.93	0.70-1.25	0.645	0.93	0.24- 3.61	0.911
Positive malaria	6.33	3.7-10.8	<0.001	22.34	12.3- 40.5	<0.001
Haemoglobin	0.94	0.85-1.04	0.235	0.82	0.63-1.06	0.136

**Table 3:** **demographic and laboratory characters associated with combined anti-malarial/antibiotic prescription for febrile less than 5-year-old children at Omdurman Hospital, Sudan using univariate and multivariate analyses**

**Variables**	**Univariate**			**Multivariate**		
	**OR**	**95% CI**	**P value**	**OR**	**95% CI**	**P value**
Age	1.0	1.00-1.02	0.001	1.0	1.00-1.03	0.012
Gender	1.3	0.68-2.48	0.428	0.8	0.23-2.57	0.675
Cough	4.1	1.91-8.71	<0.001	1.3	0.33-5.17	0.702
Runny nose	1.5	0.19-11.4	0.693	11.4	0.00-1.00	0.999
Breathlessness	3.6	1.26-10.26	0.016	0.65	0.09-4.21	0.649
Vomiting	0.4	0.22-0.84	0.014	0.2	0.06-0.70	0.012
Diarrhoea	1.0	0.38-2.66	0.975	1.5	0.27-8.28	0.646
Seizure	0.3	0.14-0.58	0.001	0.2	0.06-0.87	0.030
Inactivity	0.5	0.13-1.56	0.221	0.9	0.09-9.13	0.920
Leucocytes	1.0	1.01-1.00	0.008	1.0	1.00-1.00	0.003
Chest X-ray	0.9	0.60-1.42	0.720	1.05	0.62-1.78	0.863
Positive malaria	4.7	2.22-10.14	<0.001	1.5	0.42-5.23	0.549
Haemoglobin	0.9	0.77-1.02	0.097	0.8	0.64-0.94	0.010

The majority of the children, 87.4% (655/749) were prescribed an antibiotic. Antibiotic prescription was regardless of the status of malaria testing.

## Discussion

In malaria endemic areas including Sudan, the WHO and the National Malaria Programme recommended parasite-based malaria diagnosis by light microscopy or RDTs. Positive result is the only indication for anti-malarials treatment whereas negative cases “should be reassessed for other common causes of fever” [2,6,16,17].

This study investigated physicians’ adherence to malaria management guidelines in a reference setting in Khartoum, Sudan. Less than a half of all febrile children were tested by a parasite-based test for malaria though all should have been tested according to the management protocol [6]. Testing is a criterion of ACT-based management policy in addition to treatment of test-positive patients and not treating negatively tested patients [18]. Adherence to testing in this study was less than the adherence that reported (43.67% vs. 45.8%) by Abelgadir *et al.* two years ago in a cluster-sample survey including all age groups in Sudan [10]. This rate is however comparable to a Kenyan setting and indeed higher than evaluations in other countries [19-21]. The treating practitioners in this setting were physicians compared to the earlier cluster survey in Sudan and the aforementioned African facilities. Physicians might depend on their clinical sense more than testing. Nevertheless, testing was not requested at all in the majority of febrile children in this study, possibly physicians were investigating them for other causes of fever. Although most of the requested malaria tests in this study were microscopy, dual request for both microscopy and RDT was observed in a proportion of children possibly because physician were not relying on RCT for malaria diagnosis.

Although the majority of tested positive children were treated for malaria, almost 7% of them were not owing to the reluctance of physicians to treat possibly because of the low specificity of microscopy at hospitals in Sudan and their reliance on clinical judgment [22]. A better rate of adherence was reported from Uganda and Tanzania [16,17]. Prescription rate for negatively tested children in this study was far better than the 56% rate reported from Tanzania [23]. An even higher proportion of parasite negative patients receiving anti-malarial was conveyed from Burkina Faso [24]. Anti-malarial rate of prescription of 2% in non-malaria cases in this study was lower than Bottieau *et al.*, one of the favourable reports [25]. The varied reports may be attributed to different study designs, means of data collection and overemphasis on malaria diagnosis and treatment during training of health providers [26]. Furthermore, the conflicting overlap in IMCI programme and WHO guidelines for treatment of malaria may contribute to the varied prescription behavior among clinical practitioners [3,27].

The most striking was that injectable quinine was unduly used in this cohort for the treatment of 72.2% of children with non-severe malaria instead of the recommended first-line therapy, ACT. This highly discordant practice was almost double the figure reported by Abdelgadir *et al.* as 36% used injectable artemether monotherapy rather than quinine [10]. Nevertheless, stock-outs of ACT were reported in their study, a possible factor in faulty prescription. In a Kenyan survey, non-recommended treatments comprising either quinine monotherapy or a combination of anti-malarials and quinine was reported to become uncommon in children below five years of age and mainly prescribed for older children and adults where lower adherence for test positive patients was observed [28]. In one report, only 30% of patients were treated with ACT, suggesting that targeting of ACT would be poor even if microscopy was accurate. The findings may suggest that practitioners may clinically intend to rule out malaria in febrile children despite practice inappropriateness and inconsistency with Sudanese guidelines, possibly an IMCI effect on clinician’s prescription [6,27].

Almost 2.5% of patients not tested for malaria were treated by an anti-malarial in this cohort. This rate of presumptive treatment of malaria was less than the 19.2% rate reported by Zurovac *et al.* and Manyando *et al.* [28,29]. This group of practitioners might depend on their clinical sense and perhaps not trusting laboratory results, an argument that may be possibly augmented by the dual request of both RDT and microscopy for the same patient in this report or perhaps they were still following the IMCI guidelines.

Near to 88% of children in this study were prescribed an antibiotic whether or not tested positive for malaria. Njozi *et al.* found no significant difference in antibiotics co-prescription with anti-malarial between those tested positive for malaria and not tested in febrile patients in Tanzania [23]. However, Ugandan patients who received an antibiotic had lower odds of not being prescribed anti-malarials [16]. Different reports may be attributed to different policies and guidelines adopted by the health authorities in these countries.

This study however has numerous limitations. First, the hospital setting is a site of referral for sick children and thus results cannot be generalized as for all settings in the country. Second, prior antibiotic and anti-malarial treatment were not identified in this cohort as it may affect the practitioner’s prescription decision. Third, follow-up and patients outcome data was not assessed. Finally, observer bias cannot be ruled out although an expert clinician had collected the data.

## Conclusion

Despite a decade adoption of “test and treat” policy for malaria in this country, there is still poor adherence to malaria management protocol in Sudan among physicians treating children below five years of age. The poor adherence materializes in low testing rate, faulty prescription of anti-malarial to test-negative children and ominously about three quarters of children with malaria were treated with non-recommended anti-malarials. There is a high rate of antibiotic prescription, which needs further studies to clarify the drive behind such clinical behaviour. The ambiguity of different guidelines (malaria programme/IMCI) may contribute to the confusion of prescription among health workers.
